# Study on Mechanical Properties and Failure Mechanisms of Highly Filled Hydroxy-Terminated Polybutadiene Propellant under Different Tensile Loading Conditions

**DOI:** 10.3390/polym15193869

**Published:** 2023-09-24

**Authors:** Chengfeng Wu, Yingying Lu, Ming Jiang, Shaoqing Hu, Hongtao Yang, Xiaolong Fu, Hongyan Li

**Affiliations:** Xi’an Modern Chemistry Research Institute, Xi’an 710065, China

**Keywords:** highly filled HTPB propellant, mechanical properties, failure mechanisms, master curves

## Abstract

To study the mechanical properties of highly filled hydroxy-terminated polybutadiene (HTPB) propellant with 90 wt% solid fillers, the stress–strain curves of the propellant under different temperatures (−50 to 70 °C) and strain rates (0.000476 to 0.119048 s^−1^) were obtained by uniaxial tensile test. Moreover, to obtain the glass transition temperature and understand the effect of low temperatures on the mechanical properties of the propellant, DMA experiments were carried out. On this basis, the mechanical response laws of the propellant were analyzed, and the master curves of mechanical properties were established. Furthermore, the fracture features of the propellant under typical loading conditions were obtained by SEM, and the corresponding failure mechanisms were analyzed. The results show that the maximum strength decreases with increasing temperature, while the maximum elongation increases with increasing temperature at the same strain rate. The maximum tensile strength increases with increasing strain rate, while the maximum elongation decreases with increasing strain rate at the same temperature. The maximum tensile strength is lowest with a value of 0.35 MPa when the temperature is 343.15 K and the strain rate is 0.000476 s^−1^, at which time the maximum elongation reaches the highest with a value of 44%. In terms of failure mechanisms, the propellant shows no particle fracture, and the failure modes of the propellant are mainly matrix tearing and dewetting.

## 1. Introduction

The solid rocket motor (SRM) is widely used in the power systems of space launches, missiles, and other equipment because of its high energy density, simple structure, convenient maintenance, and low cost [1]. Solid propellant is the power source of SRM. During the storage, transportation, and use of the SRM, the solid propellant grain will be subjected to loads such as temperature, vibration, and impact [2,3,4]. If the load borne exceeds the maximum strength or elongation of the solid propellant, cracks may develop and expand inside the propellant grain, thus affecting the normal operation of the SRM [5]. Therefore, it is important to investigate the mechanical properties and failure mechanisms of solid propellants to ensure the structural integrity of propellant grain.

With the increasing demand for long-range capability in SRMs, improving the energy level of the propellant has gradually become the focus, and new energized composite solid propellants have gradually attracted wide attention from researchers [6]. In order to improve solid propellant energy, in addition to searching for new high energy density materials, increasing the content of solid phase energetic components in existing mature propellants can also lead to an increase in the theoretical specific impulse [7]. The highly filled solid propellant has the advantages of high specific impulse and high density, but with the increase in solid content, the microstructure of solid propellant becomes more complicated, which leads to greater uncertainty of mechanical properties of the propellant compared with the traditional composite solid propellant.

Hydroxy-terminated polybutadiene propellant is a commonly used composite solid propellant, belonging to the typical viscoelastic material. The mechanical properties of HTPB propellant are not only affected by temperature, but also related to strain rate, with obvious temperature effect and strain rate effect [8]. At present, researchers have carried out a large number of studies and analyses on the mechanical properties of HTPB propellants under different loading conditions using experimental and theoretical methods [9,10,11,12,13]. Zarei et al. [14] conducted a study on the mechanical properties of HTPB, and the results showed that the addition of polyethylene glycol (PEG) improved the compatibility of the plasticizer with the polyurethane (PU) matrices. The addition of PEG increased the modulus and tensile strength, while the addition of plasticizer decreased the modulus and increased the tensile strength and elongation. Liu et al. [15] carried out uniaxial tensile tests to characterize the mechanical response and failure behavior of high-performance HTPB propellant with 88 wt% fillers and studied the variation rule of the mechanical properties of propellants with strain rate and temperature under stretching. The results showed that the material parameters, such as Young’s modulus, tensile strength, and uniaxial tensile strain at maximum tensile stress, were extremely sensitive to both strain rate and temperature. Deng et al. [16] conducted tensile tests with 2.38 × 10^−5^–2.38 × 10^−2^ s^−1^, 12-week long term relaxation tests, and 4-week long term creep tests to analyze the yield characteristics and failure modes of HTPB at different strain rate conditions. The results showed that the mechanical properties of the propellant showed obvious rate dependence at lower strain rates, and the fracture strength and maximum elongation of the propellant decreased significantly with the decrease in strain rate. The microstructure entered an unstable state when the propellant started to yield. Dewetting at the interface between the matrix and the particles was gradually severe, developing into larger particle detachment and matrix tearing, and eventually causing macroscopic damage. Chen et al. [17] conducted standard relaxation tests and uniaxial tensile tests at different tensile rates for HTPB propellant, and the experimental results showed that the mechanical behavior of the material was related to the strain rate, and the yield stress and destructive stress had a significant rate dependence on the tensile rate. Meanwhile, the stiffness degradation and damage evolution of HTPB propellant were rate-dependent processes. Long et al. [18] carried out high strain rate mechanical tests for HTPB propellant with 86 wt% solid fillers using a split Hopkinson press bar (SHPB) and observed the microscopic damage of the propellant under loading conditions using SEM and computed tomography, and the results showed that the damage modes of the propellant mainly manifested as perforation fracture, increased porosity, and matrix tearing. Tussiwand et al. [19] investigated the fracture tests of HTPB propellant at different temperatures and showed that the damage region at the crack tip and crack opening displacement increased significantly at low temperatures.

From the above analysis, the current research on the mechanical properties and damage mechanism of HTPB propellant focuses on the 88 wt% solid fillers or less, while there is less research on highly filled (≥90 wt%) HTPB propellant, and the effects of temperature and strain rate on the mechanical properties the highly filled HTPB propellant are unknown. The study of the mechanical properties and damage mechanism of highly filled HTPB propellant under different temperature and strain rates is of great significance to ensure the structural integrity of the SRM charge, and it can provide support for the realization of miniaturization and remoteness for missiles to meet the needs of the application of high-energy propellants in weapons.

In the present paper, the mechanical properties of highly filled HTPB propellant with 90 wt% solid fillers under different temperatures (223.15–343.15 K) and tensile rates (2–500 mm/min) were studied by uniaxial tensile tests. Moreover, the glass transition temperature of the propellant was measured by DMA experiment, which is employed to study the effect of low temperatures on the mechanical properties. Meanwhile, the typical microscopic morphology of the tensile section was observed by SEM, which can analyze the failure mechanisms. On this basis, the effects of temperature and strain rate on mechanical properties of highly filled HTPB propellant are analyzed. Finally, the master curves of mechanical properties are set up to predict the mechanical properties under different loading conditions.

## 2. Experimental Section

### 2.1. Material Preparation

**Figure 1:**
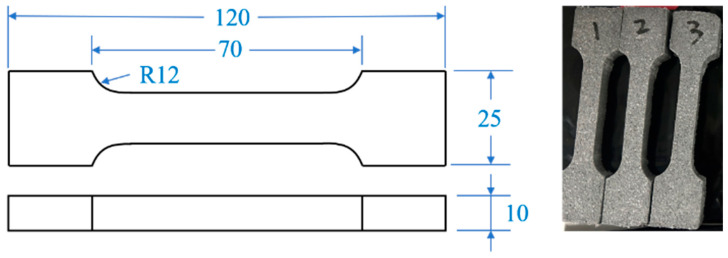
Shape and dimensions of the specimen (unit: mm).

The highly filled HTPB propellant was selected for the experimental materials. The propellant is a four-component propellant with 90 wt% solid particles, mainly consisting of non-energetic binder, non-energetic plasticizer, ammonium perchlorate (AP), cyclotetramethylene tetranitramine (HMX), aluminum powder (Al), etc. The specific mass fractions and particle sizes are shown in Table 1. Propellant specimens were prepared by slurry casting process, in which a 25 L casting process line was adopted. The main processes include raw material processing, weighing, mixing, pouring, curing, and shaping. In accordance with the standard of the People’s Republic of China GJB 770B-2005 [20], the tensile specimen was prepared into a standard dumbbell shape as shown in Figure 1, and the effective size was 70 mm × 10 mm × 10 mm. To eliminate residual stresses within the specimen, the specimen should be placed for 24 h after processing.

### 2.2. Experimental Methods

**Figure 2:**
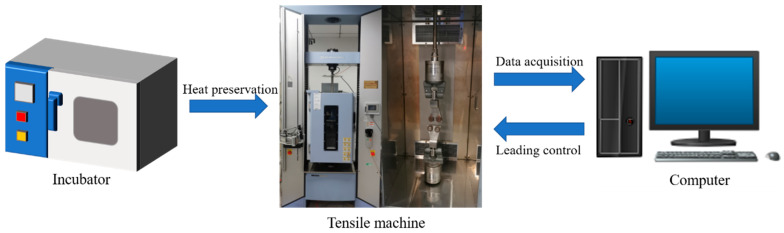
Test system.

Uniaxial tensile tests were conducted at temperatures 223.15, 253.15, 273.15, 293.15, 323.15, and 343.15 K according to the standard GJB 770B-2005 Method 413.1 of the People’s Republic of China. At each temperature, six groups of different tensile rate tests were carried out, respectively 2, 10, 20, 50, 100, and 500 mm/min. To facilitate the discussion of strain rate dependence of the propellant, the tensile rate is converted to a strain rate, and the strain rate is determined by the tensile rate and the initial length of the test section. Therefore, the corresponding strain rate is calculated as

(1) $$\dot{\varepsilon }=\frac{v}{60\times 70}$$

where $\dot{\varepsilon }$ is the strain rate, and v is the tensile rate. The six tensile rates correspond to strain rates ($\dot{\varepsilon }$) of 0.000476, 0.002381, 0.004762, 0.011905, 0.023810, and 0.119048 s^−1^, respectively. All tensile tests were carried out using an electronic universal testing machine AG-X Plus 100 that was manufactured by Shimadzu Corporation, Japan. All specimens were placed in an incubator and kept at constant temperature for 1~2 h to ensure that the internal and external temperatures of each specimen remained uniform. After the heat preservation procedure was completed, the uniaxial tensile tests were conducted. The test system consisted of a tensile machine, a computer, and an incubator, as shown in Figure 2. Five repeatability tests were conducted under each condition. The stress–strain curves are the average of the data from five tests.

To obtain the glass transition temperature, DMA experiments were carried out. The DMA 850 dynamic mechanical analysis from American TA Company was used in the test and holds the sample with a single cantilever clamp. The loading frequency was 10 Hz with an amplitude of 5 μm. The test temperature range was 153.15~273.15 K. In addition, in order to reveal the microscopic failure mechanisms, SEM was used to observe the microscopic morphology of the propellant after tensile fracture.

## 3. Results and Discussion

### 3.1. Stress–Strain Curves

**Figure 3:**
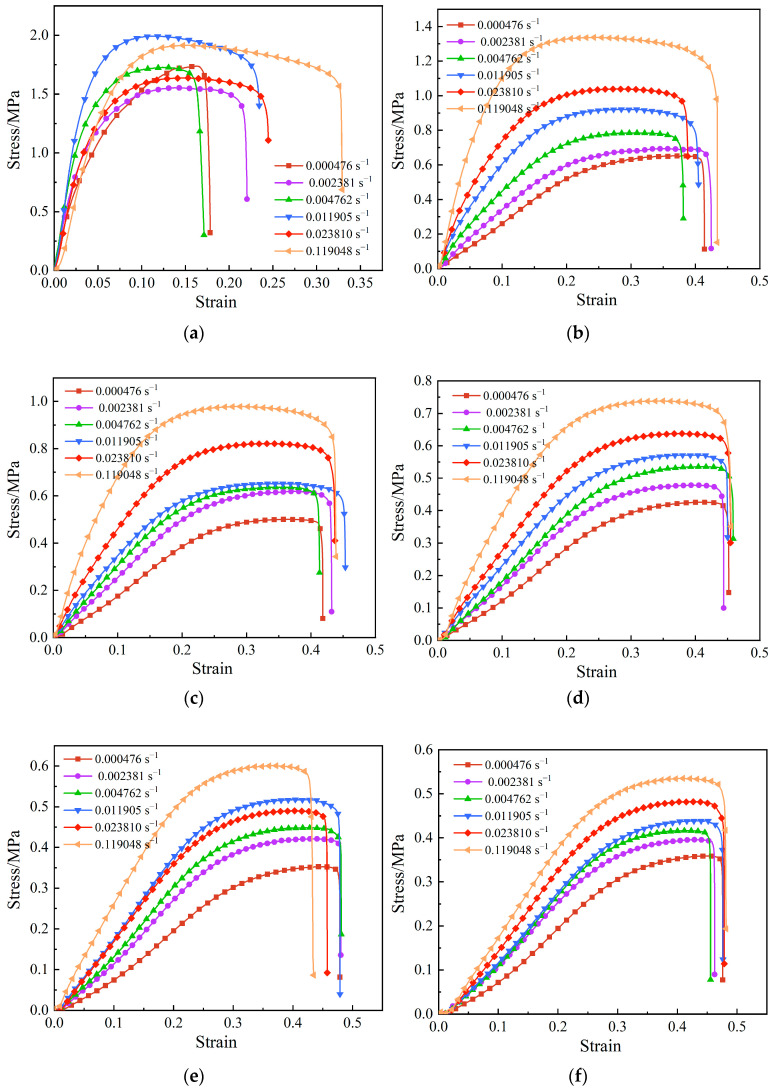
Stress–strain curves of propellant under different temperatures and strain rates. (**a**) 223.15 K. (**b**) 253.15 K. (**c**) 273.15 K. (**d**) 293.15 K. (**e**) 323.15 K. (**f**) 343.15 K.

The highly filled HTPB propellant is almost incompressible during uniaxial tension. Therefore, a slight necking phenomenon is not discussed in detail in this work. The stress–strain curves of highly filled HTPB propellant at different temperatures and strain rates are shown in Figure 3. The propellant shows typical viscoelastic behavior with significant rate dependence and temperature dependence. The reason for the viscoelastic mechanical behavior is that the binder matrix of the propellant is a polymer material in the operating temperature range [21].

**Figure 4:**
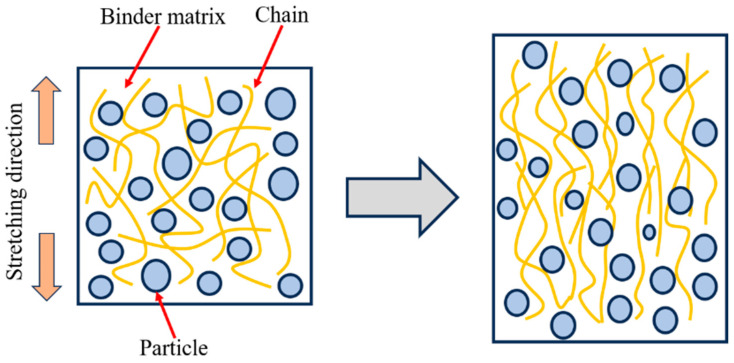
Schematic diagram of molecular chains change.

A linear random polymer, such as a binder, is a flexible long-chain polymer, and, in the highly elastic state, the moving units are individual independently rotating chain segments in the long chain. The polymer is structurally characterized by the fact that each structural unit of the molecular chain is free to rotate [22]. With proper crosslinking between molecular chains, the entire chain molecule is not easily displaced under the action of external forces, and the first motion produced is from the curled chains to the straightened state along the direction of the action force; the schematic diagram of molecular chains change is shown in Figure 4. Currently, the macromolecular chain in a state of constant thermal motion transitions from the original conformation to another conformation that is compatible with the external force, and the entropy of the system decreases. When the external force disappears, the system changes in the direction of increasing entropy, and the chain segments return to the curled state. In the process of the chain segment being straightened and returning to curl, the chain segment motion is damped as a result of the interactions between and within the molecular chains, and the equilibrium between the applied load and the occurring response cannot be reached instantaneously and takes some time to complete [23]. Therefore, the deformation of a material under the action of an external load depends on the rate of load. Furthermore, the flexibility of polymer chains arises from the rotational motion of the individual structural units that make up the molecular chain around the σ-bond that connects them, which is, in fact, a form of thermal motion of the chain, and, therefore, the viscoelastic mechanical behavior of polymers must be temperature dependent [24].

In general, the existence of several independently moving units (chain segments) between the crosslinking points within the polymer network and the nature of the individual structural units in the chain segments to rotate freely are the fundamental reasons for the viscoelastic mechanical behavior of the propellant.

### 3.2. Mechanical Response Analysis

**Figure 5:**
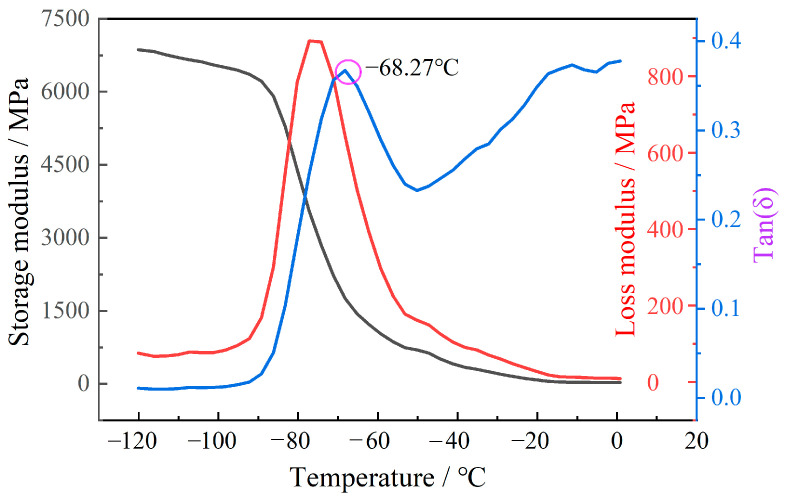
DMA curves of the highly filled HTPB propellant.

At the low temperature of 223.15 K, the mechanical response curve of the propellant exhibited no obvious regularity. However, the shape of stress–strain curve is strongly influenced by the strain rate. As the strain rate increases, the curve pattern of the platform becomes increasingly apparent. At low strain rates, the elastic section (inclined section) occupies the majority of the stress–strain curve, whereas, at high strain rates, the nonlinear section (platform section) occupies the majority of the stress–strain curve. To explain the phenomena, the DMA experiment was conducted to analyze the influence mechanisms. The variations of storage modulus ($E^{\prime }$), loss modulus ($E^{\prime \prime }$), and loss tangent (*tan δ*) with temperature are shown in Figure 5. The peak temperature of the loss tangent is picked as the glass transition temperature ($T_{g}$) [25]. As can be seen from Figure 5, the glass transition temperature of the propellant is −68 °C (205.15 K). In combination with Figure 4 and Figure 5, it can be deduced that loss modulus of the propellant is much greater when the test temperature is close to the glass transition temperature than at room temperature and high temperatures. At the moment, the molecular chains of the binder are in a frozen state; thus, the molecular chains become less flexible.

**Figure 6:**
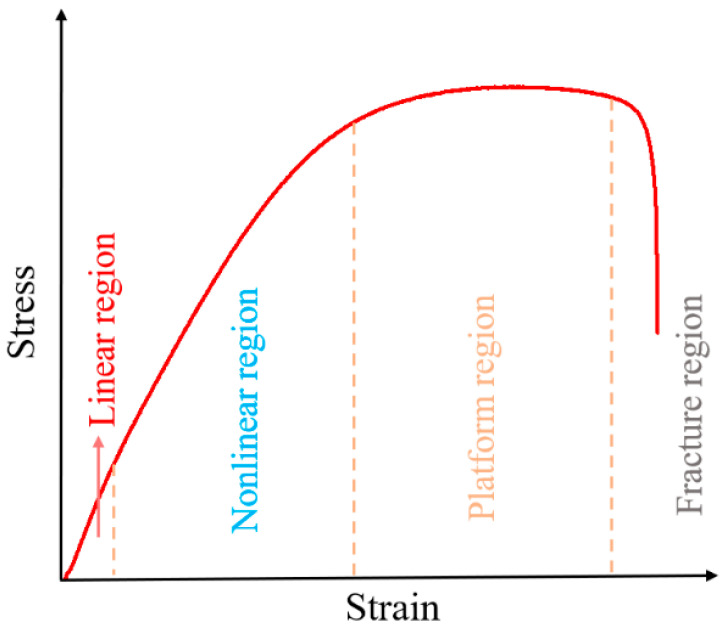
Schematic diagram of curve division.

**Figure 7:**
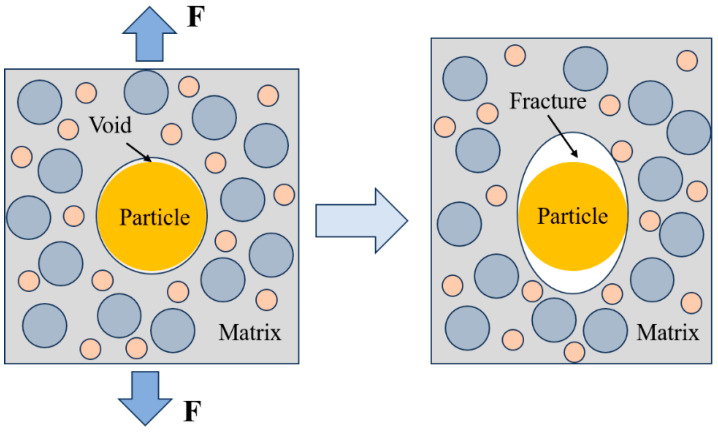
Schematic diagram of the structural evolution.

At temperatures of 253.15 K and 273.15 K, the stress–strain curves are broadly similar in shape and change slightly with the increase in strain rate. The stress–strain curve can be roughly divided into four regions, as shown in Figure 6. When the strain is low, the propellant is in an elastic state, corresponding to the linear elastic region of the stress–strain curve. As can be seen from the curve, the stress shows a linear increase with strain, and the internal structure is basically undamaged at this stage. When the strain increases to a certain value, the propellant is in the nonlinear region, at which time the trend of stress growth with strain slows down slightly, indicating that the stiffness of the material decreases. According to reference [26], microcracks or micropores begin to appear along the interface in the loading direction, that is, “dewetting” occurs in the nonlinear region. It indicates that larger particles are prone to dewetting, and the dewetting site first appears in the larger particles and larger particles aggregation area. In the larger particles aggregation area, the interaction between the particles leads to stress bridging, resulting in a stress concentration phenomenon, leading to more serious dewetting [27]. As the interface begins to take damage, the interfacial bond strength becomes weaker and the load bearing capacity decreases, hence creating the stiffness softening phenomenon. Then, as the strain reaches the platform region, the stress remains almost constant as the strain gradually increases. The displacement of the interface between the larger particles and the matrix increases, and some particles are gradually exposed, then the interfacial bonding fails; the evolutionary schematic is shown in Figure 7. As a result, the parallel stress distribution effect of each branch in the Maxwell model disappears, while the bonding around the equatorial plane of the particles is maintained, and the particles only play the role of transverse constraints on the deformation because of their own larger stiffness [28]. The interface has basically lost its load-bearing capacity, so that the stress is almost constant as the strain increases, which is the intrinsic cause of the platform region. Finally, after the strain reaches an extreme value, the stress decreases sharply with increasing strain. The matrix fully bears the load and is elongated to the point of tearing, and the propellant fracture occurs in a very short time.

**Figure 8:**
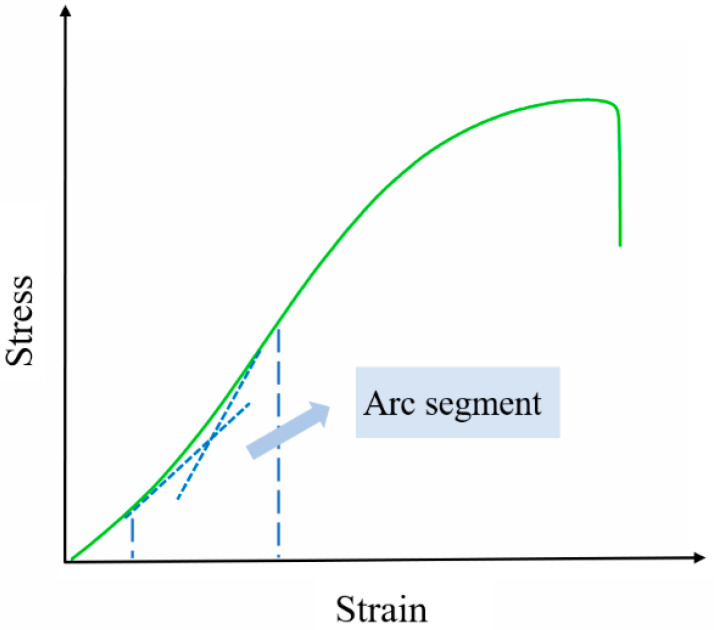
Schematic diagram of an arc segment.

At temperatures of 293.15 K and above, the shape of the stress-strain curve is less affected by the strain rate, and its shape remains almost uniform. When affected by temperature, the platform region of the curve is shortened, and the higher the temperature, the shorter the platform region. However, at low strain rates, the stress–strain curve appears to have an arc-shaped segment, as shown in Figure 8.

Few studies have been reported on the presence of such an arc segment in propellants, and similar arc segments have only been found in some rubbers [29,30]. The arc segment is generated by having a high plasticizer content in the binder matrix and can be explained by rearrangement of molecular chains according to polymers theory [31,32]. The limited stretchability of the chain network, or the capacity of molecular chains to rearrange, determines the significance of the arc segment. The flexibility of the molecular chains can be increased by plasticizer [33]. The limited stretchability increases with molecular chain flexibility. An evident arc segment and increased limited stretchability are both results of higher plasticizer content. For low strain rate, the flexible chains do not prevent each other from changing their conformation, and the curled chains can be rearranged. The curly crosslinking molecular chains are in a chaotic state at the beginning. Therefore, the curled molecular chains have a tendency towards regular arrangement under the action of external force, which corresponds to the softening arc. When the curled molecular chains take on a regular arrangement, the mechanical properties of the propellant begin to appear as a slight enhancement phenomenon with the increase in strain, which corresponds to the strengthening arc [34]. While the strain exceeds the chain extension threshold, the mechanical response will be normal.

### 3.3. Variation Law of Mechanical Parameters

**Figure 9:**
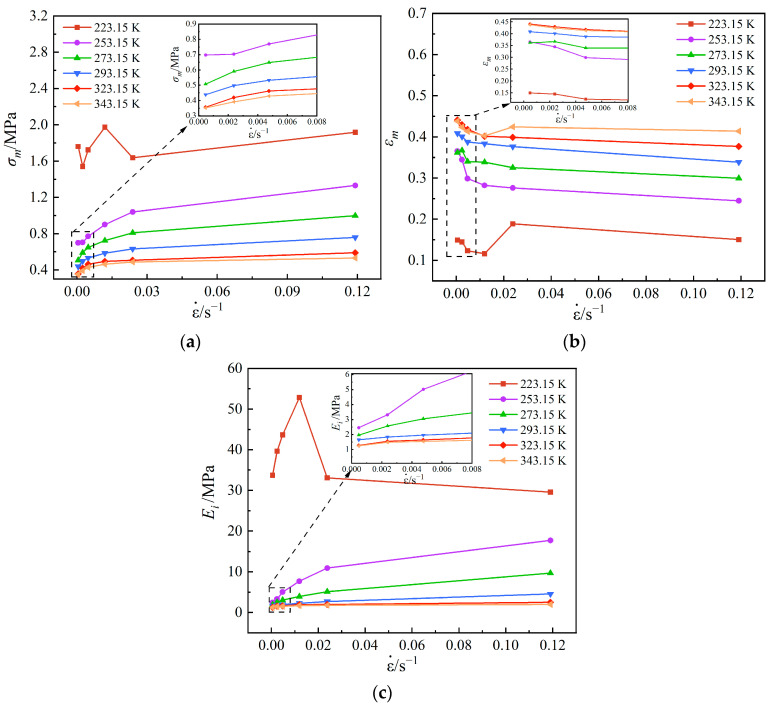
Effects of temperature and strain rate on mechanical parameters. (**a**) $\sigma_{m}$. (**b**) $\varepsilon_{m}$. (**c**) $E_{i}$.

For the strength and elongation of propellants, it is more applicable to discuss the maximum tensile strength and maximum elongation than the fracture strength and fracture elongation because the maximum tensile strength and maximum elongation are used to calculate the structural integrity of propellant grain in engineering [35,36]. If the structural strength value of propellant grain is greater than the maximum tensile strength, damage will occur inside the propellant, which is not allowed for the SRM. In order to directly reflect the influence of temperature and strain rate on the mechanical properties of the propellant, the variation of maximum tensile strength ($\sigma_{m}$), maximum elongation ($\varepsilon_{m}$) and initial elastic modulus ($E_{i}$) of the propellant with temperature and strain rate are shown in Figure 9. The slope of the linear section of the stress–strain curve is defined as the initial elastic modulus.

From Figure 9a, it can be discovered that the maximum tensile strength increases with increasing strain rate at the same temperature. The reason for this phenomenon is that the propagation of microcracks not only needs to reach the corresponding stress, but also needs a certain time. Compared with low strain rate, the occurrence of damage is relatively delayed for high strain rate; thus, the strength of the propellant is improved. Moreover, the lower the temperature, the more obvious the increasing trend, but the change law is different at the low temperature of 223.15 K. The maximum tensile strength shows a trend of first increasing and then decreasing at 223.15 K, reaching a maximum value at the strain rate of 0.011905 s^−1^, under which the tensile strength value is 1.97 MPa. In addition, the higher the temperature, the lower the maximum tensile strength at the same strain rate. The maximum tensile strength is lowest with a value of 0.35 MPa when the temperature is 343.15 K, and the strain rate is 0.000476 s^−1^.

From Figure 9b, it can be seen from the curve that the maximum elongation roughly shows a decreasing trend with increasing strain rate at the same temperature, but, at low temperatures 223.15 K and 343.15 K, the conditions do not strictly follow this law and exhibit a greater dispersion, showing similar change trend at the two temperatures. The reason for this phenomenon may be the higher solid fillers of the propellant, which do not closely obey the viscoelastic properties under severe conditions compared to the general propellant. In addition, the maximum elongation increases with increasing temperature at the same strain rate. The maximum elongation reaches the highest with a value of 44% when the temperature is 343.15 K, and the strain rate is 0.000476 s^−1^.

From Figure 9c, it can be found that the variation law of initial modulus with temperature and strain rate is almost the same as that of maximum tensile strength with temperature and strain rate. Also, the initial modulus tends to increase and then decrease with the increase in strain rate at the low temperature of 223.15 K and reaches an extreme value at 0.011905 s^−1^, where the elastic modulus is 52.85 MPa.

Based on the above analysis, it can be deduced that the mechanical behavior of the propellant at high temperature is equivalent to that at low strain rate, and the mechanical behavior of propellant at low temperature is equivalent to that at high strain rate, which also verifies the time–temperature equivalence of the propellant. Furthermore, the maximum tensile strength and maximum elongation have an opposite variation law. At the same temperature, the maximum tensile strength increases with increasing strain rate, while the maximum elongation decreases with increasing strain rate. At the same strain rate, the maximum tensile strength decreases with increasing temperature, while the maximum elongation increases with increasing temperature. Therefore, the propellant formulation design in engineering should take both properties into consideration and select the formulation with the optimal mechanical parameters for casting propellant grain.

### 3.4. Master Curves of Mechanical Properties

In general, structural integrity of propellant grain can be conveniently analyzed using mechanical parameter master curves. For instance, the master curve of the maximum strength is mainly used to predict the hazardous conditions during axial loading and ignition boost, and the master curve of the maximum elongation is mainly used to analyze the safety factor of propellant grain during solidification and cooling [37].

According to the characteristics of viscoelastic materials, increasing the rate of load is equivalent to decreasing the temperature, which is the time–temperature equivalence principle [38]. Based on this equivalence principle, researchers have proposed a folded data processing method. That is, the data measured in some specified time range and temperatures range are integrated into a curve, which is called the “Master curve”. When processing the data measured at different temperatures and rates into master curves, the key step is to stack the curves measured at each temperature by moving them in a certain direction (the reference temperature). The amount to be moved by each curve is specified by a factor with a dimension of 1, called the time–temperature conversion factor and denoted by $\alpha_{T}$, which is defined as follows:

(2) $$\alpha_{T}=\frac{t_{T}}{t_{0}}$$

where, $t_{T}$ is the time required to observe a phenomenon in the experiment when the temperature is *T*; $t_{0}$ is the time it takes for the same phenomenon to be observed at temperature *T*_0_.

To facilitate the understanding of the time–temperature equivalence principle, the W.L.F. equation was proposed by Williams, Landel, and Ferry. Therefore, the W.L.F. equation [39] was used to fit the time–temperature conversion factor at different temperatures according to the mechanical properties at different temperatures and strain rates, as shown in Equation (3).

(3) $$\mathrm{lg}\left(\alpha_{T}\right)=\frac{C_{1}\left(T-T_{0}\right)}{C_{2}+\left(T-T_{0}\right)}$$

where, *C*_1_ and *C*_2_ are the material constants; *T*_0_ is the reference temperature. The selection of reference temperature has a great influence on the accuracy of the master curve fitting, and the reference temperature is generally selected at about 60 K above the glass transition temperature; thus, *T*_0_ is chosen as 273.15 K. The values of *C*_1_ and *C*_2_ are −8.13 and 157.45, respectively.

According to the values obtained in Equation (3), the equivalent time for different temperatures and strain rates at the reference temperature is calculated, and the expression is shown in Equation (3) [33].

(4) $$\mathrm{lg}\left(\frac{1}{60\dot{\varepsilon }\alpha_{T}}\right)=\mathrm{lg}\left(\frac{1}{60\dot{\varepsilon }}\right)-\mathrm{lg}\left(\alpha_{T}\right)$$

**Figure 10:**
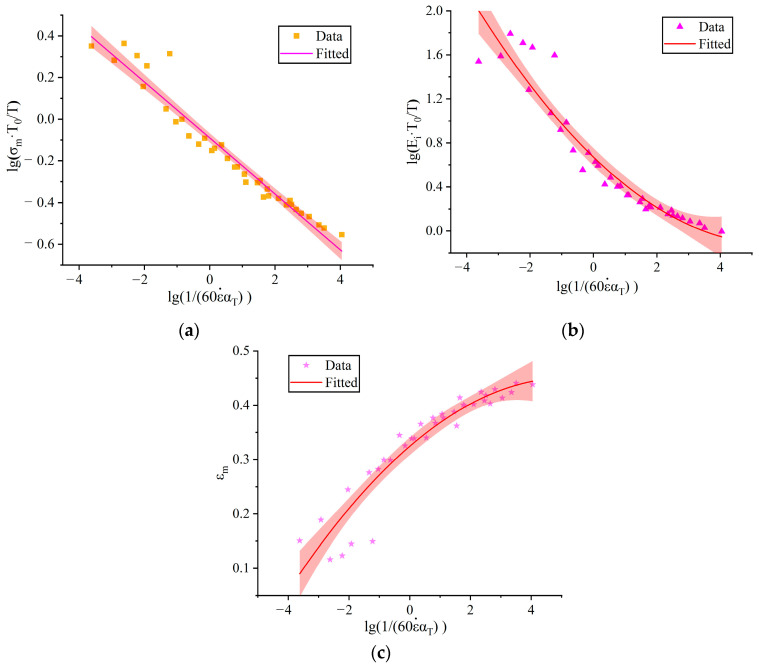
Master curves of mechanical properties of the propellant. (**a**) $\sigma_{m}$; (**b**) $E_{i}$; (**c**) $\varepsilon_{m}$.

To draw the master curve, it is necessary to select the appropriate coordinate division, and the scatter plots at different temperatures and strain rates are depicted in the coordinate diagram with $\mathrm{lg}\left(1/60\dot{\varepsilon }\alpha_{T}\right)$ as the abscissa and $lg(\sigma_{m}\cdot T_{0}/T)$, $lg(E_{i}\cdot T_{0}/T)$ and $\varepsilon_{m}$ as the ordinate, respectively. The scatter plots are then fitted to smooth curves using regression analysis, which are the respective master curves, as shown in Figure 10.

The function relationship of the master curves was obtained by fitting the scatter plots with different functions, as shown in Table 2. From the analysis in Figure 6 and Table 2, the main curve of the maximum tensile strength showed a linear decreasing trend. The main curve of the initial elastic modulus showed a quadratic function decreasing trend. The main curve of the maximum elongation showed a quadratic upward trend.

### 3.5. Analysis of Failure Mechanisms

Due to the large number of tensile tests carried out, representative loading conditions were selected here to analyze the failure mechanism. The typical temperatures selected are −50, 20, and 70 °C, and typical tensile rates are 2 and 500 mm/min.

**Figure 11:**
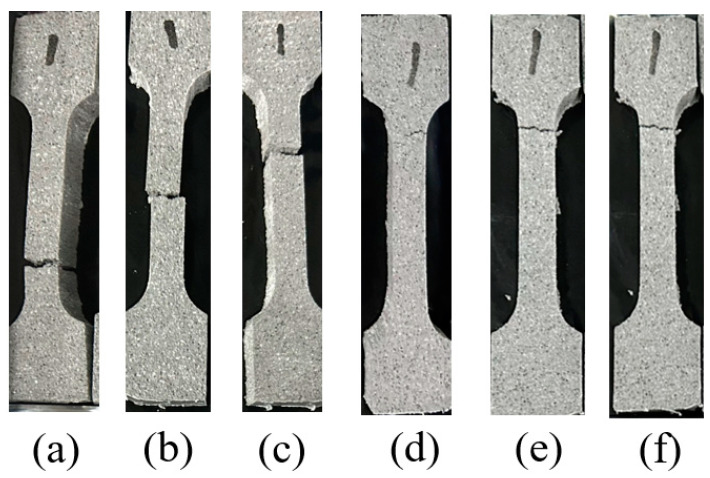
Macroscopic morphology of specimens under typical loading conditions: (**a**) −50 °C, 2 mm/min. (**b**) −50 °C, 500 mm/min. (**c**) 20 °C, 2 mm/min. (**d**) 20 °C, 500 mm/min. (**e**) 70 °C, 2 mm/min. (**f**) 70 °C, 500 mm/min.

The macroscopic morphology of the propellant specimens after tensile fracture under typical loads is shown in Figure 11. From the figure, it can be seen that the locations of fractures appearing in the specimen under different loading are not regular, but the fracture surfaces are perpendicular to the tensile direction, indicating that the uniaxial tensile fracture criterion follows the maximum positive stress criterion (the first strength theory) [40]. This criterion assumes that the fracture of the material is caused by the maximum tensile stress.

**Figure 12:**
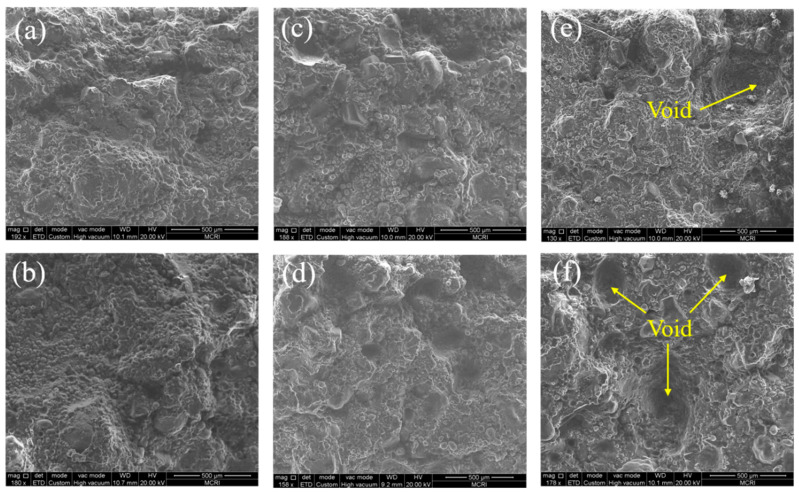
SEM images of propellant under typic loading conditions: (**a**) −50 °C, 2 mm/min. (**b**) −50 °C, 500 mm/min. (**c**) 20 °C, 2 mm/min. (**d**) 20 °C, 500 mm/min. (**e**) 70 °C, 2 mm/min. (**f**) 70 °C, 500 mm/min.

The damage of solid propellant is mainly caused by three conditions: particle fracture, matrix tearing, and interfacial damage (dewetting) [41]. In the actual failure process, the three damage modes are not individual, but various damage modes interact with each other and are coupled, often forming a complex damage mechanism. In order to clearly understand the influence of typic loading on the failure mechanisms, the cross-sections of the specimens after tensile fracture were characterized by SEM. The SEM images of the fractured section of the propellant under typical temperature (−50, 20, 70 °C) and tensile rate (2, 500 mm/min) are shown in Figure 12. As can be seen from Figure 12, the microscopic fracture morphology of propellant formed under different loads is different, which indicates that the failure mechanisms of the propellant are different under different strain rates.

From Figure 12a,b, it can be seen that, at a low temperature of −50 °C, the propellant cross section is relatively uneven and there is no void produced by particle falling off. Therefore, it can be inferred that the damage mode of the propellant at low temperatures is matrix tearing. From Figure 12c,d, it can be seen that, at room temperature 20 °C, dewetting occurs at the interface and small voids appear, so it is considered that the damage mode of the propellant at room temperature is the coexistence of matrix tearing and dewetting. As can be seen from Figure 12e,f, voids left by the ejection of larger particles appear in the propellant section, showing a typical “dewetting” feature at a high temperature of 70 °C.

At low strain rates, the propellant mostly suffers localized “dewetting” damage, which is characterized by a limited number of damaged interfaces but a significant extent of damage evolution. When the strain rate is low, the dewetting of propellant starts through the growth of microcracks and micropores at the interface. Subsequently, the damaged interfaces gradually expand and merge, with most of the damage concentrated around the larger particles. The interface experiences a rapid evolution of localized damage when the strain rate increases gradually. Nevertheless, when the strain rate increases significantly, the damage is not only confined to the surroundings of larger particles, but more damage is generated at the interface. In situations when the strain rate is increased, the dewetting of the interface has a delayed response, resulting in an increased number of interfaces that bear the load throughout the loading process. This phenomenon somewhat hinders the evolution of “dewetting” damage in the localized region of the propellant. Hence, it can be shown that the propellant interface exhibits a significant number of micropores when subjected to high strain rates. However, it is noteworthy that the extent of localized damage remains relatively low.

In addition, the number of voids increases with the increase in strain rate at the same temperature, which indicates that the particles produce more serious dewetting. Overall, the highly filled HTPB propellant shows no particle fracture, so it is concluded that the failure mechanisms of the propellant are mainly matrix tearing and dewetting.

## 4. Conclusions

In the present study, the uniaxial tensile mechanical properties and failure mechanisms of highly filled HTPB propellant under different loading conditions were investigated. The results of this study lead to the following conclusions.

The quasi-static mechanical properties of a highly filled HTPB propellant were investigated by uniaxial tensile testing. The results show that the maximum strength decreases with increasing temperature, while the maximum elongation increases with increasing temperature at the same strain rate. The maximum tensile strength increases with increasing strain rate, while the maximum elongation decreases with increasing strain rate at the same temperature. Furthermore, the maximum tensile strength is lowest with a value of 0.35 MPa when the temperature is 343.15 K and the strain rate is 0.000476 s^−1^, at which time the maximum elongation reaches the highest with a value of 44%.

It can be detected from SEM images that the tensile fracture damage modes of highly filled HTPB propellant are different at different loading conditions. The damage mode at low temperature is matrix tearing, the damage mode at high temperature is dewetting, and the damage mode at room temperature is the coexistence of matrix tearing and dewetting. With the increase in strain rate, the phenomenon of dewetting is more obvious. Overall, the highly filled HTPB propellant shows no particle fracture, and the failure mechanisms of the propellant are mainly matrix tearing and dewetting.

The master curve of highly filled HTPB propellant mechanical properties was established, which can be used to predict the maximum tensile strength of propellant under different strain rates in a wide temperature range of −50~70 °C, provide data support for the analysis of the structural integrity of propellant charges, and lay the foundation for the practical application of the highly filled HTPB propellant.

## Figures and Tables

**Table 1:** Proportion of propellant components.

Components	Mass Fraction	Particle Size
AP	48%	50~300 μm
HMX	22%	100~150 μm
Al	20%	5~20 μm
HTPB	9%	-
Other	1%	-

**Table 2:** Fitting equation of the master curves of mechanical properties.

Mechanical Parameters	Fitting Equation	R^2^
$\sigma_{m}$	$\mathrm{lg}\left(\sigma_{m}\cdot T_{0}/T\right)=-0.08975-0.13423\times \mathrm{lg}\left(1/60\dot{\varepsilon }\alpha_{T}\right)$	0.95
$E_{i}$	$\mathrm{lg}\left(E_{i}\cdot T_{0}/T\right)=0.067269-0.2786\times \mathrm{lg}\left(1/60\dot{\varepsilon }\alpha_{T}\right)+0.02447\times {\left[\mathrm{lg}\left(1/60\dot{\varepsilon }\alpha_{T}\right)\right]}^{2}$	0.91
$\varepsilon_{m}$	$\varepsilon_{m}=0.32439+0.04816\times \mathrm{lg}\left(1/60\dot{\varepsilon }\alpha_{T}\right)-0.0045\times {\left[\mathrm{lg}\left(1/60\dot{\varepsilon }\alpha_{T}\right)\right]}^{2}$	0.89

## Data Availability

Data will be made available on request.
